# Preoperative Imaging of Colorectal Liver Metastases After Neoadjuvant Chemotherapy: A Meta-Analysis

**DOI:** 10.1245/s10434-012-2300-z

**Published:** 2012-03-07

**Authors:** Charlotte S. van Kessel, Constantinus F.M. Buckens, Maurice A.A.J. van den Bosch, Maarten S. van Leeuwen, Richard van Hillegersberg, Helena M. Verkooijen

**Affiliations:** 1 Department of Surgery, University Medical Center Utrecht, Utrecht, The Netherlands; 2 Department of Radiology, University Medical Center Utrecht, Utrecht, The Netherlands; 3 Julius Center for Health Sciences and Primary Care, University Medical Centre Utrecht, Utrecht, The Netherlands

## Abstract

**Background:**

Chemotherapy treatment induces parenchymal changes that potentially affect imaging of CRLM. The purpose of this meta-analysis was to provide values of diagnostic performance of magnetic resonance imaging (MRI), computed tomography (CT), fluorodeoxyglucose positron emission tomography (FDG-PET), and FDG-PET/CT for preoperative detection of colorectal liver metastases (CRLM) in patients treated with neoadjuvant chemotherapy.

**Methods:**

A comprehensive search was performed for original articles published from inception to 2011 assessing diagnostic performance of MRI, CT, FDG-PET, or FDG-PET/CT for preoperative evaluation of CRLM following chemotherapy. Intraoperative findings and/or histology were used as reference standard. For each imaging modality we calculated pooled sensitivities for patients who received neoadjuvant chemotherapy as well as for chemonaive patients, defined as number of malignant lesions detected divided by number of malignant lesions as confirmed by the reference standard.

**Results:**

A total of 11 papers, comprising 223 patients with 906 lesions, were included. Substantial variation in study design, patient characteristics, imaging features, and reference tests was observed. Pooled sensitivity estimates of MRI, CT, FDG-PET, and FDG-PET/CT were 85.7% (69.7–94.0%), 69.9% (65.6–73.9%), 54.5% (46.7–62.1%), and 51.7% (37.8–65.4%), respectively. In chemonaive patients, sensitivity rates were 80.5% (67.0–89.4%) for CT, 81.3% (64.1–91.4%) for FDG-PET, and 71.0% (64.3–76.9%) for FDG-PET/CT. Specificity could not be calculated because of non-reporting of “true negative lesions.”

**Conclusion:**

In the neoadjuvant setting, MRI appears to be the most appropriate imaging modality for preoperative assessment of patients with CRLM. CT is the second-best diagnostic modality and should be used in the absence of MRI. Diagnostic accuracy of FDG-PET and PET-CT is strongly affected by chemotherapy.

One in two colorectal cancer patients develop liver metastases at some point during their disease.^1^,^2^ The only potentially curative option for these patients is surgical resection of their colorectal liver metastases (CRLM), after which 5 years survival probabilities of 25–58% can be achieved.^3^–^5^ Still, 80–85% of CRLM patients are not eligible for liver surgery because of extensive intrahepatic metastatic lesions or the presence of extrahepatic disease.^6^ Neoadjuvant chemotherapy is increasingly applied with the aim to downsize tumors in patients with initially unresectable disease to attain a resectable situation.^7^–^9^ Around 15–20% of these patients have their tumors rendered resectable following neoadjuvant chemotherapy and show similar survival rates as patients with initially resectable tumors.^7^,^10^,^11^

Accurate imaging of the liver following neoadjuvant chemotherapy is crucial for optimal selection of patients eligible for surgical resection. However, neoadjuvant chemotherapy may impair lesion detection and underestimate lesion size, as a result of the occurrence of intraparenchymal changes.^12^–^15^ As a result, patients whose tumors were considered resectable on preoperative imaging may turn out to have unresectable tumors during surgery.

Different imaging modalities are used in clinical practice for preoperative imaging of liver metastases. In the absence of neoadjuvant chemotherapy, contrast-enhanced computed tomography (CE-CT) and contrast-enhanced magnetic resonance imaging (CE-MRI) have been shown to be accurate diagnostic tools for preoperative imaging of CRLMs, with sensitivity rates varying from 60 to 90%.^16^–^19^ Fluorodeoxyglucose positron emission tomography (FDG-PET) may not be very informative on the anatomical location of intrahepatic lesions, but is highly sensitive for detection of intrahepatic lesions as well as extrahepatic disease.^20^,^21^ In an attempt to maintain high sensitivity while improving anatomical localization, CT and FDG-PET have now been combined into FDG-PET/CT.^21^

In the neoadjuvant setting, however, scientific evidence on the accuracy of the various imaging modalities for preoperative imaging of CRLMs is limited and ambiguous. We conducted a systematic review and meta-analysis of the literature in order to identify the optimal imaging modality for preoperative evaluation of patients with CRLM treated with neoadjuvant chemotherapy.

## Materials and Methods

Search strategy and collection of data were performed according to the guidelines of preferred reporting items for systematic reviews and meta-analyzes (PRISMA) 2009.^22^

### Data Sources and Searches

A comprehensive literature search was performed from inception to May 2011 by one observer (C.K.) for articles assessing the diagnostic accuracy of CT, MRI, FDG-PET, or FDG-PET/CT for preoperative evaluation of CRLM after neoadjuvant chemotherapy. The literature search was performed in MEDLINE and EMBASE and included synonyms for CRLM (e.g., CRLM, hepatic metastases), chemotherapy (e.g., chemotherapy, neoadjuvant treatment), and the different imaging modalities (e.g., computed tomography, CT, magnetic resonance imaging, MRI, positron emission tomography, FDG-PET, FDG-PET/CT, PET-CT). In addition, we searched reference lists of included full text articles.

#### Study Selection

Our search targeted articles based on the following inclusion criteria: patients were diagnosed with initially unresectable CRLM, patients should have been treated with neoadjuvant chemotherapy for downsizing in order to render their tumors resectable, patients were intended to undergo liver surgery, patients underwent post-chemotherapy and pre-operative imaging of the liver, and papers should present original data. Review articles, letters, comments, case reports (*n* ≤ 10), and animal studies were eliminated. Screening on title and abstract was initially performed using the aforementioned selection criteria. Of the papers that were found eligible based on title and abstract screening, full text was reviewed to further decide on suitability for inclusion in this study.

#### Quality Assessment and Data Extraction

Two observers (C.K. and H.M.V.) independently performed a critical appraisal of the remaining full text articles and extracted relevant data using a standardized form. After independent review was performed by both authors, a consensus reading was performed to discuss any disagreements in order to come to a final conclusion.

**Fig. 1:**
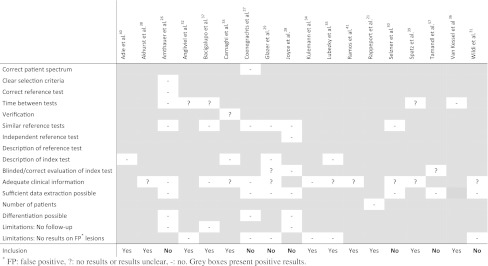
Results of critical appraisal

For each study, we extracted basic information on year of publication, characteristics of the study population (age, male-to-female ratio, site of primary tumor, proportion of patients treated with chemotherapy), and study design. Quality of the studies was quantified with a modified version of the quality assessment of diagnostic accuracy studies (QUADAS) tool of which four items were eliminated (i.e., irrelevant because of the inclusion and exclusion criteria) and four items were added (Fig. 1).^23^

The imaging technique of each study was recorded. For studies using CT, data on use of contrast material, amount of iodine, system type, slice collimation, and imaging phases (multiple or single-phase) were assessed. For MRI, the magnetic field strength, use of contrast material, sequences, and slice collimation were recorded. For FDG-PET system type, tracer specifics, scanning time, and duration of fasting time were extracted, and for FDG-PET/CT features similar to those for FDG-PET and CT were obtained.

To ensure adequate assessment of lesion detection, assessment had to be performed by a radiologist. Studies where information on lesion detection was extracted from hospital records (and not from the actual images) were excluded. To verify the presence of CRLM, we used a composite reference standard, consisting of (1) follow-up imaging for patients who did not undergo surgery, (2) intraoperative palpation, intraoperative ultrasound, and follow-up in patients who underwent surgical exploration without resection (preoperative unresectable situation), and (3) histological examination of the surgical specimen in patients who underwent surgical resection.

Total numbers of benign and malignant lesions as detected by imaging were extracted. Similarly, we extracted the total number of benign and malignant lesions detected by the reference standard. In order to determine the diagnostic performance of each imaging modality, the number of true positive, false positive, true negative, and false negative results were extracted from the article or calculated from the data (if possible). All parameters were recorded on a lesion level, for patients treated with and without neoadjuvant chemotherapy. Because of the paucity of studies reporting data on a patient level, we deemed calculating endpoints on a per-patient level to be not justifiable.

True positive lesions were defined as malignant lesions diagnosed on imaging and confirmed by the reference standard (i.e., follow-up imaging, preoperative US and palpation, or histology). False positive lesions were defined as lesions diagnosed as malignant on imaging that turned out to be benign by the reference standard. False negative lesions were defined as lesions characterized as benign or missed by imaging that turned out to be malignant based on the reference standard.

We were not able to extract data on true negative lesions, as none of the articles reported data about the detection of benign lesions that were confirmed by the reference standard (true negatives). Sensitivity was calculated as true positive lesions/(false negative lesions + true positive lesions).

#### Data Analysis

Sensitivities were calculated for each imaging modality (CT, PET-CT, FDG-PET, and MRI) and separately for patients who had received chemotherapy and those who had not. Sensitivities were logit-transformed to improve an approximate normal distribution and then pooled. Only outcomes from the same modality and with the same chemotherapy treatment status were combined. The *I* ^2^ heterogeneity statistic (estimated proportion of unexplained interstudy variance) was used to assess whether random or fixed effects were appropriate for pooling, with a 25% threshold chosen above which to apply random effects.^24^,^25^ Antilogit transformations of the resulting (pooled) sensitivities were obtained. Putatively explanatory study and population factors were assessed using mixed-effects meta-regression. Funnel plots were generated to test for publication bias. Because of the absence of reported numbers of “true negative” and “false positive” lesions we were unable to calculate specificity.

## Results

**Fig. 2:**
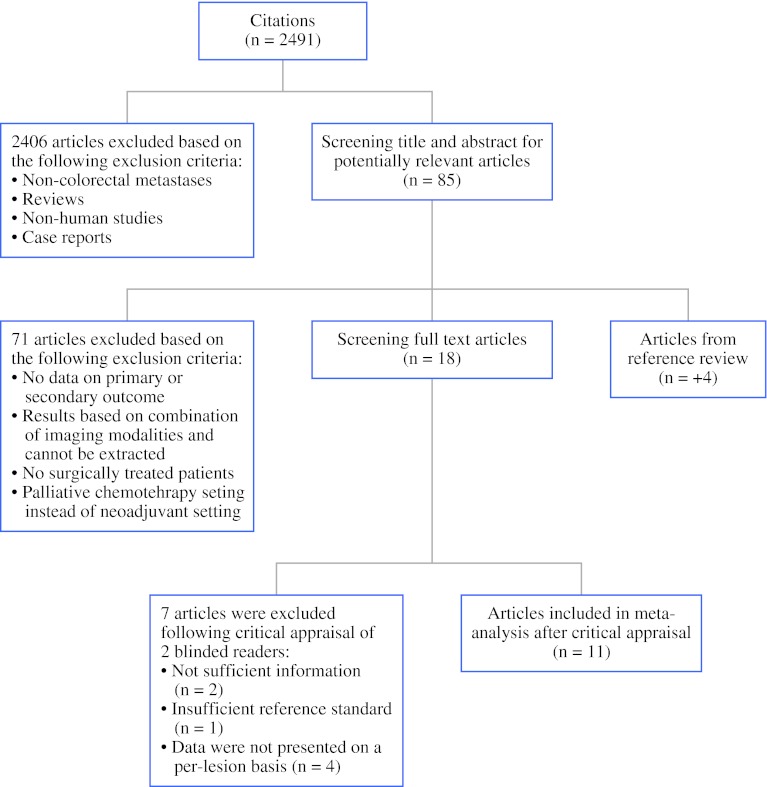
Flowchart showing the multistep process of identifying articles that were suitable for this meta-analysis for evaluation of CRLM after neoadjuvant chemotherapy

The literature search resulted in 2,491 unique references, 85 of which were potentially eligible for inclusion based on their title and/or abstract. Cross-referencing of these papers yielded four additional articles. Full text screening resulted in exclusion of another 71 articles. The remaining 18 articles met all inclusion criteria and were selected for critical appraisal (Fig. 2).

### Critical Appraisal and Study Description

Critical appraisal of the 18 articles by two observers led to exclusion of another 7 papers, because: patients receiving neoadjuvant chemotherapy could not be distinguished from patients without chemotherapy (*n* = 2), data quality was poor [i.e., retrospective data collection of CT data from hospital files without re-evaluation of the images (*n* = 1)], data were not presented on a per-lesion basis (*n* = 4).^26^–^31^ Thus, a total of 11 articles were included in our meta-analysis.

All studies were published within the last 10 years. Of the 11 studies, 6 were prospective cohort studies; the remaining 5 articles were retrospective cohort studies. Critical appraisal of quality showed that most articles adequately described in “Materials and Methods” (Fig. 1). All studies applied lesion mapping to ensure correct lesion comparison between preoperative imaging and the reference standard. A total of 906 lesions in 223 patients treated with neoadjuvant chemotherapy and 450 lesions in 265 chemonaive patients were included. Distribution of lesions detected by the different reference standards was as follows: of the patients treated with neoadjuvant chemotherapy, 835 of the 906 lesions (91.2%) were confirmed by intraoperative ultrasound followed by resection (histology), and 71 lesions (8.8%) were confirmed by follow-up imaging only. All 835 lesions in chemonaive patients were confirmed by intraoperative ultrasound followed by resection (histology). Baseline characteristics of these 11 studies are presented in Table 1.

**Table 1:** Baseline characteristics of the studies that were included in this meta-analysis

Article	Pub. date	Imaging technique	No. of patients	No. of patients with CTx	No. of lesions	No. of lesions with CTx	M/F	Mean age (yrs)^a^	Synchr/metachr mets	No. of colon/no. of rectal cancers
Akhurst et al.^38^	2005	FDG-PET	42	13	110	41	21/21	61 (30–78)	nd	nd
Lubezky et al.^35^	2007	CT + FDG-PET	75	48	155	122	53/22	nd	nd	51/24
Rappeport et al.^21^	2007	FDG-PET	35	4	71	14	16/19	62 (33–74)	nd	nd
Carnaghi et al.^33^	2007	CT + FDG-PET	19	19	65	65	12/7	61 (41–79)	12/7	14/5
Ramos et al.^41^	2008	PET-CT	63	17	125	70	41/22	62 (38–78)	31/32	nd
Angliviel et al.^32^	2009	CT	92	30	270	204	33/59	nd	49/43	32/60
Adie et al.^40^	2009	PET-CT	74	21	232	87	50/24	64 (–)	nd	nd
Bacigalupo et al.^37^	2009	MRI + FDG-PET	19	19	136	136	11/8	61 (28–74)	nd	nd
Spatz et al.^39^	2010	FDG-PET	34	17	62	37	27/7	64 (28–82)	nd	27/7
Kulemann et al.^34^	2010	CT + MRI	20	20	51	51	12/8	64 (52–77)	nd	nd
van Kessel et al.^36^	2011	CT + MRI	15	15	79	79	5/10	60 (48–71)	nd	nd
Total			488	223	1,356	906	542/344	62 (28–82)		

*nd* not defined in the article

^a^Numbers in parentheses are ranges

#### Imaging Features and Evaluation

Computed tomography was evaluated in five studies.^32^–^36^ A helical system was used in one study, multidetector CT systems in two studies, a single slice system in one study, and one study did not report on the system used. Intravenous contrast was used in four studies (non-ionic agents in three studies), and one study did not report on the use of a contrast agent. Also, four studies reported on using multiple phase imaging. Section thickness (2–5 mm) was described in three studies.

Magnetic resonance imaging was evaluated in three studies.^34^,^36^,^37^ Of these, two studies used 1.5 Tesla systems, and one study combined 1.5 and 3.0 Tesla systems. Gadolinium-based contrast was used for dynamic scanning in one study, and two studies used superparamagnetic iron oxides (SPIOs) or other liver-specific contrast.

Accuracy of FDG-PET was assessed in six studies.^21^,^33^, ^35^,^37^–^39^ All studies used different scanning systems; three studies reported a fasting period of 4–6 h. The amount of tracer varied between 250 and 666 MBq. All six studies reported an interval between contrast injection and scanning of 60–120 min. Only two studies reported on the duration of scanning time (3–4 min per bed position in 6–7 bed positions).

The effectiveness of PET-CT for detection of CRLM was assessed in two studies.^40^,^41^ Only limited information on the PET-CT protocol was reported. Both studies used Discovery LS PET/CT systems (GE Medical Systems). One study reported on a fasting duration of 4–6 h and use of 370 MBq FDG. One study did not report on the CT protocol. The other study performed a single-phase non-contrast-enhanced CT prior to the PET scan.

All studies used intraoperative ultrasound to confirm the presence of CRLM and to detect any additional lesions, and all studies considered histological examination to be the primary reference standard. In patients who turned out to have unresectable disease during surgery, intraoperative ultrasound was used as reference standard. In three studies, which included patients who were deemed unresectable on preoperative evaluation, follow-up imaging was used to confirm the presence of CRLM in the non-operable patients by assessing lesion growth over time.^30^,^36^,^37^

**Fig. 3:**
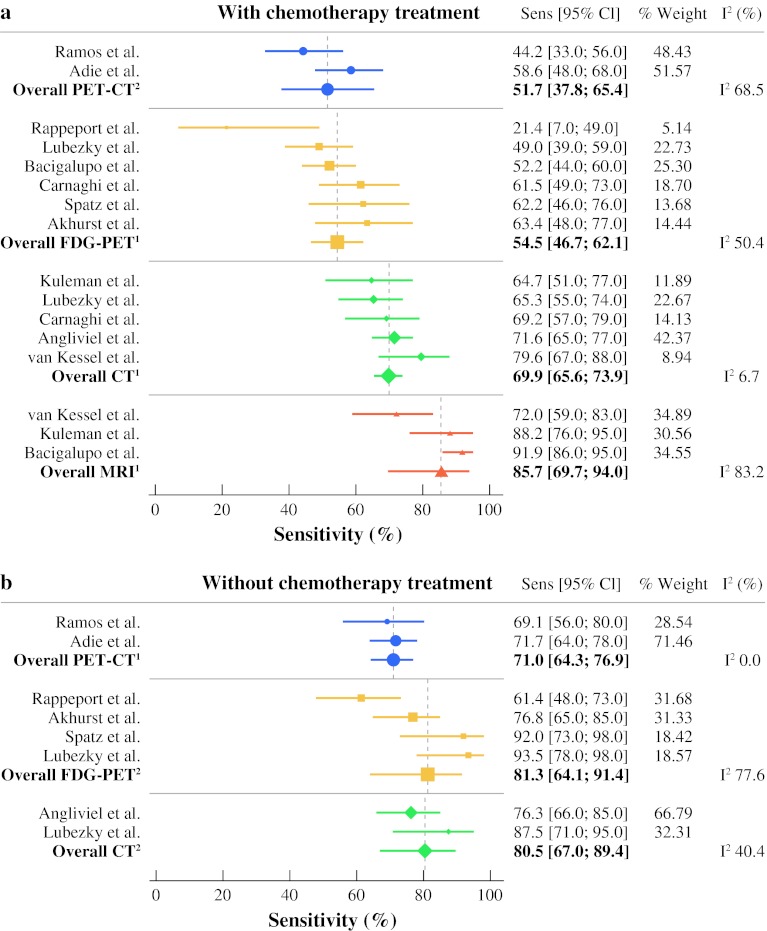
Forest plots showing pooled sensitivities for MRI, CT, FDG-PET, and PET-CT on a lesion level. **a** Results are displayed for the patients who received neoadjuvant chemotherapy. **b** Results are displayed for patients without neoadjuvant chemotherapy

For patients treated with neoadjuvant chemotherapy, relevant data were available for 3, 5, 6, and 2 studies on MRI, CT, FDG-PET, and PET-CT, respectively. A heterogeneous distribution of sensitivities was observed for MRI, FDG-PET, and PET-CT (*I* ^2^ > 25%), while the sensitivity distribution of CT was homogeneous (*I* ^2^ = 6.75%). Pooled sensitivity estimates were 85.7% (69.7–94.0%) for MRI, 69.9% (65.6–73.9%) for CT, 54.5% (46.7–62.1%) for FDG-PET, and 51.7% (37.8–65.4%) for PET-CT (Fig. 3).

In the chemotherapy-naive setting, relevant data were available for 2, 4, and 2 studies on CT, FDG-PET, and PET-CT, respectively. Homogeneous sensitivity distribution was seen for PET-CT (*I* ^2^ = 0%), while CT and FDG-PET showed heterogeneous sensitivity distribution. Pooled sensitivities were 80.5% (67.0–98.4%) for CT, 81.3% (64.1–91.4%) for FDG-PET, and 71.0% (64.3–76.9%) for PET-CT. No studies reported on diagnostic performance of MRI in chemonaive patients.

Mixed-effect meta-regression analysis showed that differences in sensitivity rates for the various imaging modalities were not explained by study and population variables (i.e., age, gender, synchronous/metachronous CRLM).

#### Publication Bias

Visual inspection of the funnel plots did not show any signs of gross publication bias.^42^

## Discussion

Accurate preoperative imaging of CRLM is crucial for optimal selection of patients suitable for surgery. With this meta-analysis we show that according to the currently available evidence, MRI is the preferable imaging modality for evaluation of CRLM in the neoadjuvant setting, with a pooled sensitivity of 85.7%. However, it has to be taken into account that this estimate is based on a limited number of studies and that SPIO contrast agents were used in two of these diagnostic studies, while this contrast agent is rarely used in current clinical practice because of significant side effects and high costs. Furthermore, this meta-analysis showed that in the absence of MRI, CT is the best alternative with a pooled sensitivity of 69.9%. Both FDG-PET and PET-CT, which perform rather well in chemonaive liver metastases, have a low diagnostic performance in the neoadjuvant setting.

The negative impact of neoadjuvant chemotherapy on the diagnostic performance of the various imaging techniques was most obvious for FDG-PET and PET-CT, where sensitivity rates decreased from 81.3 and 71.0%, respectively, in chemonaive patients to 54.5 and 51.7%, respectively, in patients treated with chemotherapy. This was a rather unexpected finding, especially for PET-CT. This may be explained by, firstly, both PET-CT studies included in this meta-analysis were of small sample size and, secondly, because sensitivity results in chemonaive patients were also rather low in both studies. These results might improve in future studies as PET-CT has been introduced and optimized during the past years. One reason behind the chemotherapy-induced decrease in diagnostic performance of FDG-PET and PET-CT may include induced necrosis, which may give initially solid metastases a more cystic appearance. MRI and CT might still visualize these lesions during the arterial phase in the form of rim enhancement. On FDG-PET, however, there is no FDG-uptake in areas with necrosis, and therefore lesions are not visualized.^43^ Another explanation could be that neoadjuvant chemotherapy reduces the average size of CRLM, and FDG-PET is known to have a lower sensitivity for detection of subcentimeter lesions than CT or MRI.^18^,^44^,^45^ In addition, chemotherapy reduces metabolic activity of cancer cells [in particular the activity of the glycolytic hexokinase enzyme (GLUT-1 transporter) that collects FDG], which may hamper visualization of the lesions on PET.^38^ CT and MRI imaging is not affected by this phenomenon.

The diagnostic performance of CT was also affected by neoadjuvant chemotherapy, albeit to a lesser extent than that of FDG-PET and PET-CT. A mechanism behind this observation might be that neoadjuvant chemotherapy causes changes of the liver parenchyma, such as steatosis (irinotecan and 5-FU) or sinusoidal obstruction (oxaliplatin).^12^,^15^,^46^ For CT it has been shown that neoadjuvant chemotherapy results in a lower density of the liver parenchyma and less contrast enhancement, leading to a decreased liver-to-lesion contrast, thereby hindering the detection, characterization, and delineation of lesions.^14^,^32^

Our meta-analysis shows that MRI has the highest diagnostic performance in the neoadjuvant setting. However, two of the three studies that were identified used SPIO contrast agents. SPIO agents have been replaced largely by gadolinium-based agents since SPIOs are costly and require an extensive scanning time, and side effects frequently occur.^47^ Currently, gadolinium-based agents and liver-specific agents such as Gd-EOB-DTPA (Primovist) are used in routine clinical care for evaluation of CRLM. The sensitivity of gadolinium-based agents for detection of CRLM in chemotherapy-naive patients is about 80%.^18^ However, only one study assessed the use of gadolinium-enhanced MRI for detection of CRLM after chemotherapy, and in this study a sensitivity of 72.2% was observed.^36^ Data on the performance of liver-specific agents such as Gd-EOB-DTPA (Primovist) for detection of CRLM after chemotherapy are lacking, although in nontreated patients high sensitivities for detection of CRLM up to 95% have been reported.^48^–^51^ As the diagnostic performance of MRI is strongly dependent on the type of contrast agent used, further research on this imaging modality using the currently available contrast agents in patients with chemotherapy treatment is warranted. These studies might show even better diagnostic performance for MRI in the neoadjuvant setting than was observed in this meta-analysis.

We acknowledge that our meta-analysis suffers from several limitations. No numbers of true negative and false positive lesions could be extracted reliably in the majority of studies, and therefore specificity could not be calculated. However, accurate characterization of benign lesions is essential, as overestimation of liver lesions (i.e., rating a benign lesion as malignant) can lead to the incorrect decision of omitting surgery. In a previous study, our group has shown MRI to be superior to CT in differentiating between CRLM and benign lesions as CT was more likely to overestimate the number of CRLM.^36^ Most articles did not incorporate follow-up data in their reference standard, which may have led to overestimation of sensitivity rates, as lesions that were missed in the non-operated liver segments are unaccounted for.^52^ However, studies reporting on patients who did not receive resection following chemotherapy because of unresectability, but did receive follow-up imaging to confirm the presence of CRLM, were included in this meta-analysis.

Although there are numerous studies assessing patients with metastatic colorectal cancer, only 11 studies were eligible for inclusion in this meta-analysis. This was mainly because the majority of studies assessing diagnostic accuracy of imaging modalities for detection of CRLM included chemonaive patients only or did not separately report on patients with and without chemotherapy.

Finally, there were insufficient data to perform a meta-analysis on an individual patient level. Still, per-patient-based data are not pertinent for determining the diagnostic value of the different imaging modalities in preoperative evaluation of CRLM. However, in addition to lesion-based sensitivity data, data on resection outcome by imaging modality would be of great clinical relevance. Although we consider this to be a limitation of this meta-analysis, it is a reflection of the currently available evidence as data on which imaging modality results in the best resection outcome are currently lacking in the neoadjuvant setting. Therefore, a trial comparing contrast-enhanced CT and gadolinium-enhanced or liver-specific contrast-enhanced MRI would be appropriate. The design of this trial should not only allow for assessment of lesion detection and characterization, but also for lesion localization and resection strategy, in order to determine which imaging modality most accurately determines treatment strategy.

## Conclusion

The results of this meta-analysis suggest that MRI is the most appropriate imaging modality for preoperative detection of CRLM in patients treated with neoadjuvant chemotherapy. CT is the second-best diagnostic modality and should be used in the absence of MRI. FDG-PET and PET-CT, which perform well for imaging of chemonaive CRLM patients, should be avoided for preoperative evaluation of patients in the neoadjuvant setting.
